# An Objective Non-Reference Metric Based on Arimoto Entropy for Assessing the Quality of Fused Images

**DOI:** 10.3390/e21090879

**Published:** 2019-09-10

**Authors:** Bicao Li, Runchuan Li, Zhoufeng Liu, Chunlei Li, Zongmin Wang

**Affiliations:** 1 School of Information engineering, Zhengzhou University, Zhengzhou 450001, China; 2 School of Electronic and Information Engineering, Zhongyuan University of Technology, Zhengzhou 450007, China (Z.L.) (C.L.); 3 Cooperative Innovation Center of Internet Healthcare, Zhengzhou University, Zhengzhou 450000, China; 4 Research Institute of Industrial Technology, Zhengzhou University, Zhengzhou 450000, China

**Keywords:** Arimoto entropy, fusion metric, multi-focus images, non-reference

## Abstract

In the technologies, increasing attention is being paid to image fusion; nevertheless, how to objectively assess the quality of fused images and the performance of different fusion algorithms is of significance. In this paper, we propose a novel objective non-reference measure for evaluating image fusion. This metric employs the properties of Arimoto entropy, which is a generalization of Shannon entropy, measuring the amount of information that the fusion image contains about two input images. Preliminary experiments on multi-focus images and multi-modal images using the average fusion algorithm, contrast pyramid, principal component analysis, laplacian pyramid, guided filtering and discrete cosine transform have been implemented. In addition, a comparison has been conducted with other relevant quality metrics of image fusion such as mutual information, normalized mutual information, Tsallis divergence and the Petrovic measure. The experimental results illustrate that our presented metric correlates better with the subjective criteria of these fused images.

## 1. Introduction

Image fusion is a significant technology in the field of digital image processing. The fusion between the different images accounts for the process of integrating two or multiple source images into a new image. Currently, many fusion algorithms have been presented. Additionally, a number of encouraging fusion results have also been provided. It is essential to evaluate the quality of fused image and the performance of fusion methods. In developing a taxonomy of image fusion metrics, it is common to make a basic distinction between subjective and objective assessments. A subjective [1] assessment (i.e., human visual perception) is reliable, but it is also tedious, time-consuming and expensive. Therefore, there is a high expectation for objective evaluation metrics to evaluate the quality of fused images.

In recent years, researchers have proposed many measures for the objective assessment of image quality, such as the mean square error (MSE), the Peak Signal to Noise Ratio (PSNR), and the structural similarity index measure (SSIM) [2]. Wang modeled any image distortion as a combination of three factors: luminance distortion, correlation losing and contrast distortion, and proposed a universal image quality index (UIQI) [3]. Although this index did not exploit the human visual system, the experimental results demonstrate that it performs essentially better than other metrics. However, a reference image must be constructed as the ground-truth for the evaluation of the image fusion performance and a comparison with the experimental results by these metrics [4]. Nevertheless, it is in fact usually difficult to define an accurate reference value in image fusion evaluation.

Therefore, it is of great significance to study an objective fusion evaluation measurement without a reference value. Several objective fusion metrics have been presented, where the ground truth data is not assumed [5]. Petrovic applied an operator based on the Sobel edge to generate the edge magnitude, along with an orientation information, employed to assess the fused image [6]. Local measures were utilized by Piella in order to measure how well the salient information from two input images was present in the output images, and they introduced a new objective quality evaluation metric without reference [7]. Xydeas [8] introduced an assessment framework of image fusion exploiting gradient information representation, in which information contributions of every sensor, the gain of fusion, the loss of information and artifacts in image fusion were quantified, and an in-depth analysis of the fusion performance was provided. Chen and Varshney [9] partitioned the whole image into a set of non-overlapping areas, in which the response of the human visual system (HVS) was simulated by a contrast sensitivity function (CSF), and the difference of two images in the frequency domain was modified. The similarity measures in local regions were obtained, and the weighted summation over all of these non-overlapping windows was defined as the global quality metric.

Considering human perception and the imaging characteristics of the infrared and visible images, Wang [10] divided the source images into various local regions, and the regional information similarity was evaluated for the quality of the fused image. A notion called fusion consistency was first introduced by Kotwal and Chaudhuri [11], and they suggested that the fusion methods should satisfy the consistency criterion. Several modifications for a number of existing measures were presented to efficiently quantify the progression of the image fusion. Han et al. [12] presented a fusion metric based on a multi-resolution strategy, where visual information fidelity (VIF) was employed to evaluate the performance of the image fusion objectively. In their scheme, the input and output fused images were first filtered and separated into blocks. In each block, the visual information was evaluated with and without distortion information, after which the VIF for fusion (VIFF) for every sub-band was estimated. The VIFFs of each sub-band were weighted to determine the quality metric, and the experimental results demonstrate that the novel metric fits well with the HVS and indicates the lower computation complexity. Li et al. [13] proposed a metric based on similarity, employing a model of luminance that is generally leveraged in visual psychophysics and a new contrast model that correlates better with human visual sensitivity to estimate the similarity of the output fused image and the input source images [14]. The concept of the just noticeable blur (JNB), which simulates the human visual system (HVS), was introduced and integrated into a model of probability summation, and a perceptual-based no-reference objective image sharpness/blurriness metric was presented, which can measure the relative amount of images blurriness.

Other widespread metrics to assess the quality of the fused image are based on information theory. In information theory, the most commonly-used measure is mutual information (MI), which is a measure of the amount of information that one random variable contains about another random variable [15]. An objective non-reference fusion metric exploiting the MI between the fused image and source images was proposed to evaluate the performance of image fusion by Qu et al. [16]. MI is employed to measure what amount of information was transferred from the input images to the final output images. 

Subsequently, a normalized metric based on MI was proposed by Hossny et al. [17]. On the other hand, Cvejic et al. [18] pointed out that the MI metric does not correlate well with the subjective tests of the output images, and introduced an alternative information-based metric. This metric applied Tsallis entropy, a generalization of Shannon entropy, to assess the performance of image fusion.

Inspired by prior observations, a new objective performance evaluation metric for fusing multi-focus images and multimodal images is introduced in this paper. This metric employed Arimoto entropy to assess the performance of image fusion. The presented metric is consistent with the subjective visual inspection.

## 2. Preliminaries

For one discrete stochastic variable *X*(*x*_1_, *x*_2_,…, *x_N_*), with its probability distribution *P*(*p*_1_, *p*_2_,…, *p_N_*), i.e., $\sum_{i=1}^{N}p_{i}=1$ with *p_i_* = *P*(*x* = *x_i_*) > 0, the well-known Shannon entropy for *X* is given as follows [15]:

(1) $$H(X)=-\sum_{i=1}^{N}p_{i}\log p_{i}$$

The Shannon entropy shown in Equation (1) is employed to measure the average uncertainty contained in one stochastic variable. In other words, it provides the information amount included in this random variable. Given another random variable *Y*, with probability distributions *Q*(*q*_1_, *q*_2_,…, *q_N_*), the Kullback–Leibler (KL) divergence between *X* and *Y* is described as:

(2) $$D\left(X||Y\right)=\sum_{i=1}^{N}p_{i}\log\frac{p_{i}}{q_{i}}$$

Mutual information is a particular case of Equation (2), defined as the KL divergence between the joint probability and the product of two marginal probabilities:

(3) $$I\left(x,y\right)=\sum_{x,y}p\left(x,y\right)\log\frac{p\left(x,y\right)}{p\left(x\right)\cdot p\left(y\right)}$$

where the joint and marginal probabilities can be estimated through the simple normalization of joint and marginal histograms. Qu et al. exploited the MI of the fused image and two input images to construct a metric for the image fusion performance. Let *A* and *B* be the two input images to be fused, and let *F* denote the fused image. The metric for the image fusion quality in [16] was given as:

(4) M(A,B;F)=I(F,A)+I(F,B)

This metric represents the total amount of information that *F* contains about *A* and *B*. In Equation (4), *I*(*F*, *A*) and *I*(*F*, *B*) represent the MI measures between *F* and *A*, *B*, respectively.

## 3. Proposed Metric

However, Cvejic et al. pointed that the MI fusion metric has significant disadvantages and does not correlate well with the subjective evaluation when using the average method and other fusion metrics. The Arimoto entropy, which is considered as a generalized definition of the classical Shannon entropy and which has similar properties to Shannon entropy, was presented by Arimoto in [19], along with its properties further reported in [20,21]. The Arimoto entropy of one random variable is defined as:

(5) $$A_{\alpha }(X)=\frac{\alpha }{\alpha -1}\left[1-{\left(\sum_{i=1}^{N}p_{i}^{\alpha }\right)}^{\frac{1}{\alpha }}\right]\;\alpha >0,\alpha \neq 1$$

It has been shown that when *α*→1, using L’Hopital’s rule, the limitation of Arimoto entropy is equivalent to the classical Shannon entropy. It is reported by Boekee et al. [20] that Arimoto entropy is a nonnegative and pseudo-additivity entropy. Therefore, we obtain:

(6) $$A_{\alpha }(X)\geq 0\;\alpha >0,\alpha \neq 1$$

and

(7) $$A_{\alpha }(X,Y)=A_{\alpha }(X)+A_{\alpha }(Y)-\frac{\alpha -1}{\alpha }A_{\alpha }(X)A_{\alpha }(Y)\;\alpha >0,\alpha \neq 1$$

where *X* and *Y* represent two independent stochastic variables, *A*_α_(*X, Y*) denotes the joint Arimoto entropy of the two variables, with *A*_α_(*X*) and *A*_α_(*Y*) being the marginal Arimoto entropy for *X* and *Y*, respectively, over the interval of *α*∈(0,1)⋃(1,+∞). Some significant properties of Arimoto entropy were deduced by Boekee et al. [20]; here, several useful properties are only exhibited as follows:

- Concavity:
  (8) $$A_{\alpha }(tX_{1}+(1-t)X_{2})\geq tA_{\alpha }(X_{1})+(1-t)A_{\alpha }(X_{2})\;t\in (0,1),\alpha >0,\alpha \neq 1$$
- Symmetry:
  (9) $$A_{\alpha }\left(\cdots ,p_{i},\cdots p_{j}\cdots \right)=A_{\alpha }\left(\cdots ,p_{j},\cdots p_{i}\cdots \right)$$
- Upper bound:
  (10) $$A_{\alpha }\left(p_{1},p_{2},\cdots ,p_{N}\right)\leq A_{\alpha }\left(\frac{1}{N},\frac{1}{N},\cdots ,\frac{1}{N}\right)=\frac{\alpha }{\alpha -1}\left[1-N^{\frac{1-\alpha }{\alpha }}\right]$$

See [20] for the detailed proof of these properties. The Kullback–Leibler divergence is a measure of the distance between two discrete random variable distributions *P* and *Q*. The Kullback–Leibler divergence based on Shannon entropy is given by $\sum_{i}p_{i}\log p_{i}/q_{i}$, which measures the distance of the two probability distributions *p_i_* and *q_i_*. The Kullback–Leibler distance was generalized using the definition of the Tsallis entropy [22]. The Arimoto entropy has similar properties to the Tsallis entropy, and it is also a generalization of the Shannon entropy. Inspired by the definition of the Tsallis divergence in the work of [22] and based on the definition of the Arimoto entropy shown in Equation (5), we introduce the definition of the Arimoto divergence using the KL distance of the Arimoto entropy:

(11) $$D_{A}\left(X||Y\right)=\frac{\alpha }{1-\alpha }\left[1-{\left(\sum_{i=1}^{N}\frac{p_{i}^{\alpha }}{q_{i}^{\alpha -1}}\right)}^{\frac{1}{\alpha }}\right]\;\alpha >0,\alpha \neq 1$$

According to the pseudo-additivity property and similar to the Tsallis mutual information (MI), the MI of the Arimoto entropy is given by:

(12) $$I_{\alpha }\left(X,Y\right)=A_{\alpha }(X)+A_{\alpha }(Y)-\frac{\alpha -1}{\alpha }A_{\alpha }(X)A_{\alpha }(Y)-A_{\alpha }(X,Y)\;\alpha >0,\alpha \neq 1$$

Consequently, the similarity based on the Arimoto divergence between the fused image *F* and input image *A* is calculated as:

(13) $$I_{\alpha }\left(F,A\right)=\frac{\alpha }{1-\alpha }\left[1-{\left(\sum_{i=1}^{N}\frac{p{\left(f,a\right)}^{\alpha }}{{\left(p\left(f\right)\cdot p\left(a\right)\right)}^{\alpha -1}}\right)}^{\frac{1}{\alpha }}\right]\;\alpha >0,\alpha \neq 1$$

Similarly, the similarity between *F* and *B* is given by:

(14) $$I_{\alpha }\left(F,B\right)=\frac{\alpha }{1-\alpha }\left[1-{\left(\sum_{i=1}^{N}\frac{p{\left(f,b\right)}^{\alpha }}{{\left(p\left(f\right)\cdot p\left(b\right)\right)}^{\alpha -1}}\right)}^{\frac{1}{\alpha }}\right]\;\alpha >0,\alpha \neq 1$$

The proposed metric to evaluate the performance of the image fusion is finally described as:

(15) $$M_{\alpha }(A,B;F)=I_{\alpha }\left(F,A\right)+I_{\alpha }\left(F,B\right)$$

## 4. Experimental Section and Results

To assess the performance of our presented metric, two groups of fusion experiments on multi-focus and multi-modal images are implemented using several algorithms. The experiments are carried out in MATLAB 2016a with an HP computer and Microsoft Windows 10 Operation system, as well as a 2.6 GHz and 8 GB memory PC.

### 4.1. Test Data and Fusion Methods

**Figure 1:**
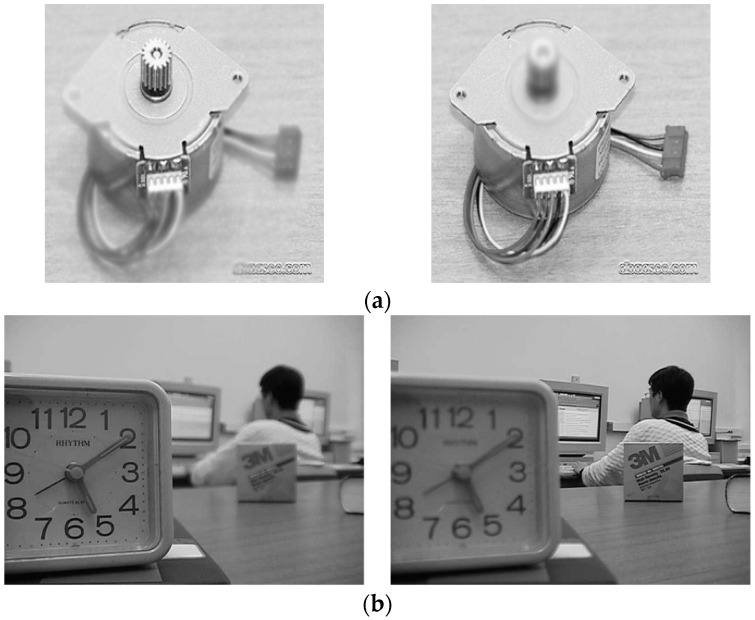
Multi-focus images. (**a**) “Gear” images, and (**b**) “Laboratory” images.

**Figure 2:**
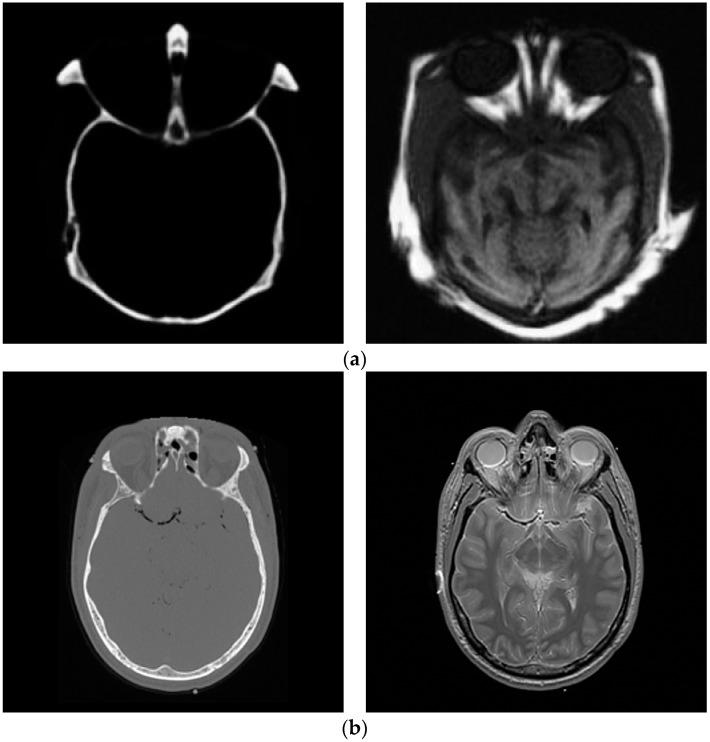
Multi-modal images, (**a**) CT and (**b**) MRI images.

Two groups of tested images, in which the first group includes two pairs of multi-focus images and the second one consists of the multi-modal images (two pairs of CT and MR images), were exploited in order to evaluate our proposed metric. The input original images adopted in our fusion experiments were taken from the website: https://github.com/sametaymaz/Multi-focus-Image-Fusion-Dataset and http://www.metapix.de/. Figure 1 and Figure 2 depict the original images used in the multi-focus and multi-modal image fusion experiments, respectively.

The dimensions of these tested images to be fused are displayed in Table 1. To assess the final fused images and compare the performance of different fusion methods, several methods for image fusion were exploited to carry out fusion experiments on two input images (with the identical scene), along with the final fused image obtained.

Numerous technologies of image fusion are introduced in many literatures. Instead of recent methods, six classical algorithms (the simple averaging method, Contrast Pyramid [23], Principal Component Analysis [24], Discrete Cosine Transform [25], Laplacian Pyramid [26], and guided filtering [27] denoted by {‘averaging’, ‘CP’, ‘PCA’, ‘DCT’, ‘LP’ and ‘GF’}) are selected to validate the proposed metric. The reason is that we only focus on the assessment of the measure performance for image fusion and not on comparing various image fusion approaches. The averaging method is the simplest and is frequently adopted in the field of image fusion, and it is a pixel-by-pixel average of two input images. For the CP and LP methods, the original images are decomposed by filtering and the base coefficients of the last decomposition stage are selected; additionally, the fused image is reconstructed. In the PCA method, the covariance matrixes of two original images are first computed, and their eigenvalues are calculated, selected and normalized, together with the fused image that is obtained. In the DCT method, two original images are first decomposed into 8 × 8 blocks. In these blocks, the 2D DCT are computed, and the calculated transform coefficients are normalized. Combining with the means and variances of the image blocks, the inverse DCT of these blocks are computed, and the final image is constructed. The same rules for image fusion are employed for the aforementioned CP and LP approaches. The GF method decomposed an image into a base layer containing large scale variations in intensity, and a detail layer capturing small scale details.

Some detail information may be lost by the averaging method, while on the contrary, the DCT, LP and GF methods can reserve the detail information of the two input images. In conclusion, the optimal output image should largely keep the detail information from the two input images.

### 4.2. Multi-Focus Image Fusion

**Figure 3:**
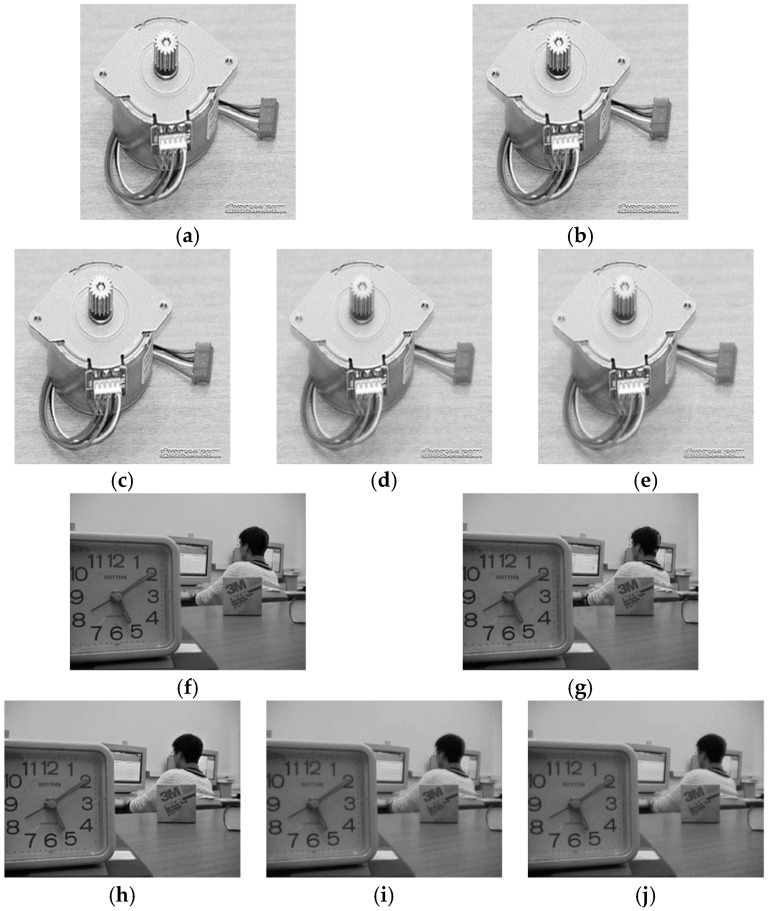
The fused images. (**a**–**e**) The fused results of the multi-focus “Gear” images using GF, DCT, CP, PCA, and average method, respectively; (**f**–**j**) The fused images of the multi-focus images obtained by the five algorithms.

The aim of image fusion is to generate more informative images that are suitable for human visual perception; namely, the final fusion images obtained by different fusion methods must fit with the human visual system (HVS). Therefore, the objective assessment should correlate well with the subjective perception. In multi-focus experiments, the fused images were acquired by applying the simple averaging, CP, PCA, DCT and GF methods, respectively. The fused images obtained by exploiting the five algorithms are exhibited in Figure 3.

It is obviously observed from Figure 3 that the best fused images for the multi-focus “Gear” and “Laboratory” images are obtained by the DCT and GF methods. In addition, the second to the fifth of the fusion performance for the “Gear” images are GF, CP, PCA and the average method. For the “Laboratory” images, the order of the fused images is as follows: DCT, CP, PCA and the average method. In comparison with other fusion metrics, the subjective rank of the fused results by the five algorithms is tabulated in the last column of Table 2 and Table 3.

**Figure 4:**
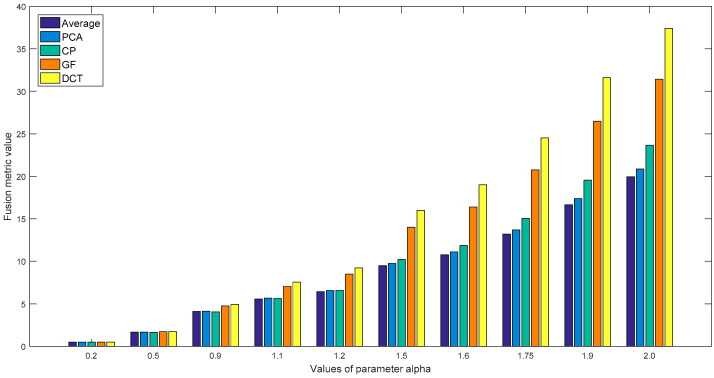
The results of the various parameter values of our metric for the “Gear” fused images of five methods.

**Figure 5:**
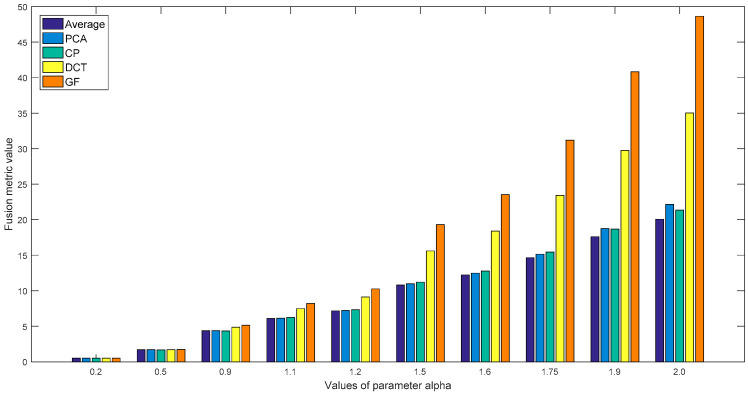
The results of the various parameter values of our metric for the “Laboratory” fused images of five methods.

To illustrate the role of the parameter α in the proposed metric, we applied our metric with various parameter values to the fused images of the five algorithms shown in Figure 3. Using ten values of the parameter α [0.2, 0.5, 0.9, 1.1, 1.2, 1.5, 1.6, 1.75, 1.9, 2.0], these experiments were carried out, and the test results of the “Gear” and “Laboratory” images are displayed in Figure 4 and Figure 5. It can be observed from these results that when the parameter values are relatively small, for example α = 0.2, 0.5, 0.9 or 1.1, it is difficult for these metric values to distinguish the fusion performance of PCA, CP and the average method. Additionally, Figure 5 shows that the quality of the fused images (α = 1.9, 2.0) by PCA and CP cannot correlate with the subjective rank. The metric values of α = 1.5, 1.6 and 1.75 provide the superior results and the corresponding assessment with the subjective rank, and these results also demonstrate that the values of the proposed metric increases as the quality of the fused images increases.

According to the aforementioned fusion experiments, we find the optimal values of α, so that the obtained metric values would correlate with the subjective performance and α was set to 1.5. For the comparison between our proposed metric and other objective measures, Table 2 and Table 3 show the metric values between these fused images and input multi-focus images calculated by the mutual information (MI) [16], normalized mutual information (NMI) [17], the Petrovic measure, Tsallis divergence [18] and the proposed method, respectively. We can see that the proposed metric provides the consistency evaluation order for the five fusion algorithms: ②①③④⑤ for the “Gear” and ①②③④⑤ for the “Laboratory” images, which are the same as the subjective rank. However, the evaluated results of other metrics do not exactly correlate with the subjective test. According to the experimental results, the proposed metric for the image fusion performance based on Arimoto entropy can correctly evaluate these fused images employing different algorithms, which also demonstrates that the presented metric is an effective evaluation measure for assessing the quality of multi-focus images.

### 4.3. Multi-Modal Image Fusion

**Figure 6:**
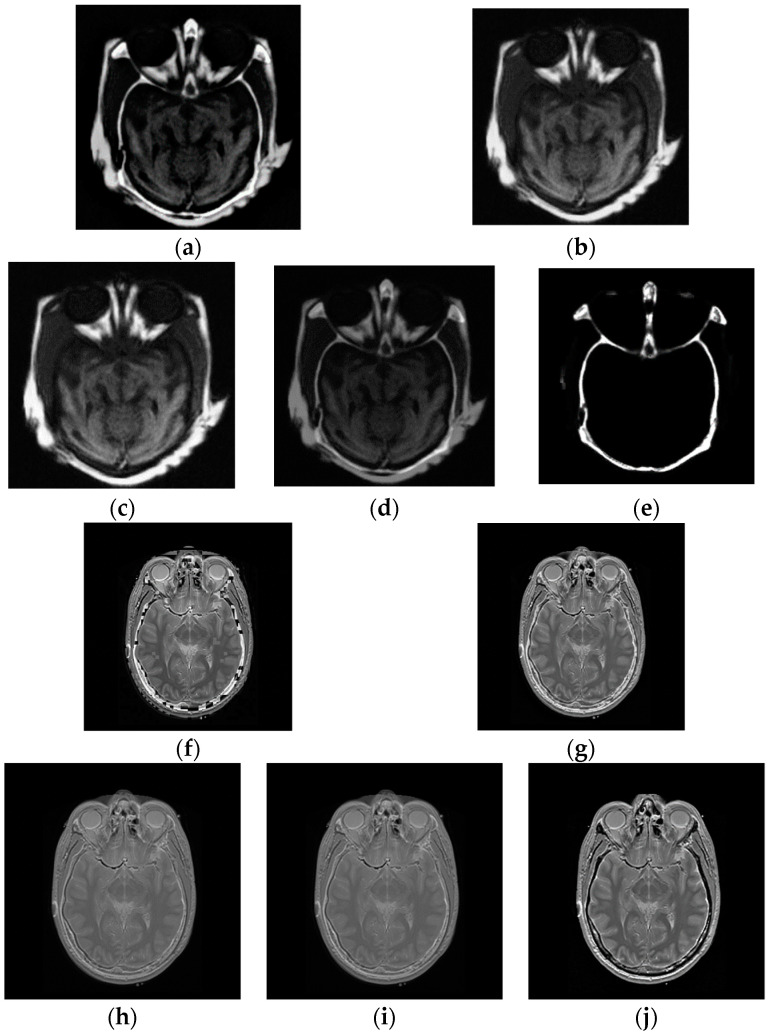
The fused images. (**a**–**e**) The fused results of the multi-modal images shown in Figure 2a using LP, PCA, DCT, average method and CP respectively; (**f**–**j**) The fused images of the multi-modal images in Figure 2b obtained by DCT, LP, average method, PCA and CP.

In multi-modal tests, the original images shown in Figure 2 are applied to generate the fused images. The fusion experiments are performed on these images using CP, PCA, DCT, LP and the average methods, and the corresponding fused results, displayed in Figure 6a–e, show the fused results for two original images in Figure 2a using five algorithms, together with Figure 6f–j giving the fused images of the two input images shown in Figure 2b.

The final fused images should both include the information of the two original images. Nonetheless, in the first test of the multi-focus images, the fused image in Figure 6e by CP fusion algorithm almost only consists of the information of the CT image and hardly includes the information of the MR image. The fused results obtained by PCA and DCT are similar, and only provide the information of the MR image instead of the CT image. The LP fusion method illustrates the information of the CT image and also displays the MR information. Likewise, for the fusion experiments of the two original images given in Figure 2b, the fused image acquired via the CP algorithm only exhibits the MR information and does not obtain the information from the CT image.

Similarly to section B, to validate the performance of our proposed metric in the fusion of multi-modal images, Table 4 and Table 5 depict the metric values between the fused images and original multi-focus images sketched in Figure 2a,b calculated by mutual information, NMI, the Petrovic measure, Tsallis divergence and the proposed method, respectively.

It is observed from Table 4 and Table 5 that the proposed metric can provide the consistency evaluation order for two groups of multi-modal image fusion adopting five fusion algorithms, which is the same as the subjective rank. However, the evaluated results of the MI, NMI, Tsallis divergence and Petrovic metric do not correlate with the subjective test. The experimental results demonstrate that the presented metric is an effective evaluation measure for the fusion performance of multi-modal images.

## 5. Conclusions

In this work, we presented a non-reference objective metric of image fusion performance based on Arimoto entropy. This metric provides an objective evaluation of the fused results acquired by the fusion methods in the absence of a reference value. The fusion experiments on multi-focus images and multi-modal images were carried out, and the results demonstrated that our presented metric can effectively assess the performance of image fusion and correlate well with subjective quality.

## Figures and Tables

**Table 1:** The dimensions of the input images.

Image	Dimension	Figure
Gear	256 × 256	Figure 1a
Laboratory	480 × 640	Figure 1b
CT & MR	256 × 256	Figure 2a
CT & MR	464 × 464	Figure 2b

**Table 2:** The evaluation results of these fused “Gear” images obtained by five algorithms applying five objective fusion metrics and the subjective rank.

	“Gear” Images					Subjective Rank
Method/Metric	MI	NMI	Petrovic	Tsallis	Proposed	
DCT	8.796①	1.319①	0.852②	24.96①	15.99①	①
GF	8.347②	1.252②	0.854①	20.97②	14.005②	②
CP	6.846⑤	1.028⑤	0.842③	13.86③	10.228③	③
PCA	6.998③	1.061③	0.794④	13.04④	9.746④	④
Average	6.92④	1.05④	0.787⑤	12.57⑤	9.475⑤	⑤

**Table 3:** The evaluation results of these fused “Laboratory” images obtained by five algorithms applying five objective fusion metrics and the subjective rank.

	“Laboratory” Images					Subjective Rank
Method/Metric	MI	NMI	Petrovic	Tsallis	Proposed	
GF	7.911②	1.133②	0.751①	30.66①	19.31①	①
DCT	8.516①	1.224①	0.742②	23.33②	15.597②	②
CP	7.018⑤	1.003⑤	0.711③	15.47③	11.218③	③
PCA	7.122③	1.027③	0.59④	15.03④	10.974④	④
Average	7.08④	1.021④	0.589⑤	14.77⑤	10.812⑤	⑤

**Table 4:** The evaluation results of the fused images obtained through four algorithms on the original images shown in Figure 2a, applying three objective fusion metrics and the subjective rank.

	CT-MR Images in Figure 2a					Subjective Rank
Method/Metric	MI	NMI	Petrovic	Tsallis	Proposed	
LP	2.564④	0.453⑤	0.729①	31.652①	19.101①	①
PCA	6.238②	0.981②	0.649③	24.408③	13.729②	②
DCT	7.028①	1.092①	0.667②	23.221④	13.15③	③
Average	5.164③	0.879③	0.42④	27.369②	12.631④	④
CP	1.635⑤	0.729④	0.253⑤	12.404⑤	9.0675⑤	⑤

**Table 5:** The evaluation results of the fused images obtained through four algorithms on the original images shown in Figure 2b, applying three objective fusion metrics and the subjective rank.

	CT-MR Images in Figure 2b					Subjective Rank
Method/Metric	MI	NMI	Petrovic	Tsallis	Proposed	
DCT	5.911①	1.12①	0.673①	27.732①	17.858①	①
LP	3.236④	0.68⑤	0.609②	13.800④	14.888②	②
Average	4.281②	0.921②	0.399④	21.573②	14.403③	③
PCA	4.071③	0.90③	0.363⑤	15.990③	11.534④	④
CP	3.088⑤	0.698④	0.566③	12.738⑤	9.536⑤	⑤
